# Power Law versus Exponential State Transition Dynamics: Application to Sleep-Wake Architecture

**DOI:** 10.1371/journal.pone.0014204

**Published:** 2010-12-02

**Authors:** Jesse Chu-Shore, M. Brandon Westover, Matt T. Bianchi

**Affiliations:** 1 Institute for Quantitative Social Science, Harvard University, Cambridge, Massachusetts, United States of America; 2 Partners Neurology, Brigham and Women's Hospital and Massachusetts General Hospital, Boston, Massachusetts, United States of America; 3 Sleep Division, Neurology Department, Massachusetts General Hospital, Boston, Massachusetts, United States of America; 4 Division of Sleep Medicine, Harvard Medical School, Boston, Massachusetts, United States of America; Harvard University, United States of America

## Abstract

**Background:**

Despite the common experience that interrupted sleep has a negative impact on waking function, the features of human sleep-wake architecture that best distinguish sleep continuity versus fragmentation remain elusive. In this regard, there is growing interest in characterizing sleep architecture using models of the temporal dynamics of sleep-wake stage transitions. In humans and other mammals, the state transitions defining sleep and wake bout durations have been described with exponential and power law models, respectively. However, sleep-wake stage distributions are often complex, and distinguishing between exponential and power law processes is not always straightforward. Although mono-exponential distributions are distinct from power law distributions, multi-exponential distributions may in fact resemble power laws by appearing linear on a log-log plot.

**Methodology/Principal Findings:**

To characterize the parameters that may allow these distributions to mimic one another, we systematically fitted multi-exponential-generated distributions with a power law model, and power law-generated distributions with multi-exponential models. We used the Kolmogorov-Smirnov method to investigate goodness of fit for the “incorrect” model over a range of parameters. The “zone of mimicry” of parameters that increased the risk of mistakenly accepting power law fitting resembled empiric time constants obtained in human sleep and wake bout distributions.

**Conclusions/Significance:**

Recognizing this uncertainty in model distinction impacts interpretation of transition dynamics (self-organizing versus probabilistic), and the generation of predictive models for clinical classification of normal and pathological sleep architecture.

## Introduction

Although it has been understood for decades that sleep is comprised of transitions among sub-stages of rapid eye movement (REM) and non-REM (NREM) sleep, whether the temporal dynamics of these transitions is important for restorative functions remains obscure. Interestingly, recent analysis demonstrates that standard metrics used to summarize sleep architecture in clinical studies (sleep efficiency, percentages of each stage) fails to identify differences in fragmentation caused by medically severe sleep apnea [1], [2]. This suggests that alternative measures are necessary to better characterize sleep architecture and its fragmentation in disease. Recent work suggests that human and animal sleep architecture dynamics can be quantified by methods emphasizing stage transition probabilities [1], [2], [3], [4], [5], [6], [7], [8]. These methods assess transitions mainly via the distribution of bout lengths of individual sleep-wake stages, which are clearly non-Gaussian. The distribution of bout lengths of sleep has been described as an exponential process in mice, rats, cats, and humans [3], [4], [6], while the distribution of wake bout lengths has been described as either exponential [6], or, more commonly, as a power law across these species [3], [4]. Some data in fact suggests that newborn rodents exhibit exponential distribution of waking bouts, which then evolves into a power law distribution as the animal matures [9]. Although a flip-flop neuronal circuit model has been proposed to control the transitions between sleep and wake (and a separate flip-flop switch for transitions between NREM and REM sleep) [10], modeling linking these neural circuits to the fine structure of sleep architecture is lacking.

Improved quantification of sleep architecture holds promise for correlating sleep disruption with daytime symptoms and different pathological causes of fragmentation. Understanding sleep-wake transitions also has implications for modeling sleep architecture dynamics. Power laws and exponentials are apparent in many aspects of biology, from molecular to system levels, but may have distinct mechanistic implications. Power law distributions are thought to arise from multiple interacting components of a complex system, and observations of such systems often follow a similar profile across multiple measurement scales (“scale-free” patterns). Examples of scale-free patterns include stock market fluctuations (similarly jagged over minutes, days, or years), pulmonary branching patterns, activity level fluctuations, and heart rate variability [11], [12], [13]. Exponential processes imply probabilistic state transitions typically governed by a constant rate of change over time. For example, state transitions in enzymes and ion channels typically exhibit exponential kinetics. The distribution of sleep-wake transitions may thus hold important clues to understanding the mechanisms underlying sleep architecture in health and disease.

We ask therefore, under what conditions one distribution is likely to be mistaken for the other in terms of fitting, and consider the effects of sample size and the parameters of the distributions using simulation methods. We hypothesize that with relatively small sample sizes (such as that which might be typical of 1–14 clinical sleep study nights), it is likely that statistical testing will yield an acceptable fit for a power law distribution, even if the true underlying model is multi-exponential, and vice versa. We further hypothesize that certain combinations of exponential distributions will be particularly susceptible to mistaken acceptance of power law fitting.

## Materials and Methods

We are primarily concerned with two distributions commonly used to describe the lengths of sleep and wake bouts. A power law function has the general form *f*(*x*)  =  *c*x^−α^, where c is a constant, and α is the “scaling factor”, that is, the slope of the line seen on a log-log plot. An exponential function has the general form *f*(*x*)  =  *a*e^(−*x*/τ)^, where *a* is the relative contribution of the given exponential component (see below), and τ is the time required for the function to decay by 63%, and thus is a measure of the rate of decay (smaller numbers indicate faster decay rates). The simulations and analysis below were performed using R.

### Kolmogorov-Smirnov test of goodness of fit

We follow Clauset et al [14] in using the Kolmogorov-Smirnov (KS) test, a non-parametric test of goodness of fit, used for assessing the probability that a sample of observations was drawn from a given population distribution (whether empirically measured or mathematically generated). In other words, the test asks if the observations in the population and the observations in the sample follow the same statistical distribution. Although the KS test can be used to compare two empirically observed samples, in our simulations we refer to the “population distribution” as the standard against which sample observations are compared.

The cumulative distribution function (CDF) is defined as the probability (*y*-axis) that an event is shorter than or equal to a given bout length (*x* axis). The KS test statistic, *d_o_*, measures the maximum vertical distance (maximum difference in cumulative probability) between the observed sample CDF and the given population CDF. The logic of the test is that if the sample CDF is drawn (statistically) from the same distribution as the population CDF, then the CDFs will be close and thus *d_o_* will be small. If *d_o_* is not small, then this is considered evidence that the sample observations are not distributed according to the population CDF under consideration [15].

This type of analysis requires a sense of how far from zero *d_o_* can be expected to fall. For most observed sample distributions, the distribution of *d_o_* is not known, and therefore must be established by simulation methods. In the parlance of hypothesis testing, the null distribution of *d_o_* should be specified, and then experimental observations can undergo comparison testing via KS. Below we establish criterion for rejecting the null hypothesis (that two distributions are not different) through numerical simulation methods (that is, drawing random variables from pre-specified population distributions).

Algorithm summary: (Supplemental Figure S1) For each combination of sample size and parameter values examined, we iterated over the following five steps 1000 times, in order to estimate a *p*-value:

1. Draw a test sample from a known distribution: either a power law random number generator, or an exponential random number generator with one, two, or three exponential components.
2. Fit the test sample to the other (incorrect) distribution and estimate the KS test statistic, *d_o_*.
3. Generate 100 reference sample datasets from random number generators with distributions defined by the fitted functions used in step 2.
4. Re-fit each of these 100 reference sample datasets to the type of distribution from which they were drawn in step 3, and estimate the test statistic for simulated data, *d_s_*. The *d_s_* values define the range of expected deviation from the distribution fitted in step 2, given its parameters and sample size.
5. Compare *d_0_* to each of the 100 values of *d_s_* from step 3, to estimate a *p*-value for the proposition that the test sample from step 1 was drawn from the distribution fitted in step 2 (*p*  =  fraction of the time *d_o_*≤*d_s_*).

The output of each iteration is a *p*-value, calculated by comparing one test sample to 100 random samples drawn from the incorrect distribution. We use *p* = 0.05 as the critical value for statistical rejection of the fitted model. Our results report the probability of failing to reject the incorrect model as the fraction of the 1000 iterations for which *p*>0.05. In the results section, each data point plotted in the figures and each entry in the tables represents the results from one run of 1000 iterations for the specified combination of parameter values and sample size.

### Fitting a power law function to samples drawn from exponential distributions

Test samples of data were randomly drawn from the sum of either two or three exponential distributions. Each test sample was fitted to a power law distribution, and the goodness of fit was evaluated by the KS method. We systematically varied the proportion of observations drawn from each exponential distribution and its decay parameter, τ (the average duration of an observation), in order to determine the goodness of fit of the power law function over a spectrum of parameter values. The number of observations included *n* = 40, *n* = 160, *n* = 320 and *n* = 640 (except in some cases to maintain equal proportions, we used *n* = 39, *n* = 159, *n* = 318 and *n* = 639).

Note that the methods of Clauset et al [14] for fitting power laws to empirical data include an estimation of a lower threshold of event duration, below which the distribution does not exhibit power law behavior. For our simulated sleep bouts, following this threshold method resulted in good but meaningless estimates of fit due to discarding a large portion of the sample data. Accordingly, we fixed the lower threshold to one epoch to standardize the comparison of fit across the parameter space as well as to guarantee that the fit of the entire sample was considered.

### Fitting exponential functions to samples drawn from a power law distribution

We generated sleep bouts as random values drawn from a power law distribution with a scaling exponent (α) of 3, each of which is referred below as a “test sample”. We set α = 3 to ensure adequate dispersion of the duration distribution given our binning routine (1 epoch bin width, all fractions rounded down). We then collected these simulated bouts into frequency-duration histograms (see below), in preparation for three separate fitting routines: a single exponential function, the sum of two exponential functions, and the sum of three exponential functions of x. For example, the form of the three-exponential function is f(*x*)  =  *a*_1_e^(−*x*/τ1)^ + *a*_2_e^(−*x*/τ2)^+ *a*_3_e^(−*x*/τ3)^, where *a*_i_ is the relative contribution of the i^th^ exponential term to the distribution, defined by the *y*-intercept of that component of the multi-exponential equation. τ is the time-invariant rate of change of the function, and is sometimes called the scaling factor of the exponential term. Although exponential functions typically contain a constant term to account for a *y*-axis offset, this parameter was forced to zero in this analysis, given that there is no biological basis for postulating a constant minimum frequency for all possible sleep durations (this constraint does not affect the arguments presented here). We also limited analysis to only decaying functions with a positive estimate of τ (such that the exponent –*x*/τ remained negative).

The shortest state defined by convention in human sleep studies is 30 seconds, or one “epoch”, which is the unit of time used in these simulations. In generating random data, we discarded values less than 1 epoch; in other words, *n* values reflect the number of draws ≥1 epoch. Although rounding does occur in clinical scoring (minimum threshold 0.5 epoch to score a stage), we did not round fractional state durations. However, the 1-epoch width of bins in our frequency-duration histograms effectively rounded down any fractional state durations, similar to Clauset et al [14]. Each simulated sample of bouts was binned, and the resulting histograms were fit using exponential models via the non-linear least-squares Levenberg-Marquardt algorithm (LMA) (http://cran.r-project.org/web/packages/minpack.lm/index.html; accessed 1/12/10). We maintain bins of constant width, rather than logarithmically increasing widths. Logarithmic binning compresses long duration events into relatively fewer bins, by comparison. As the number of observations per bin is lower for longer durations using constant bin width, this could theoretically result in longer observations carrying disproportionate weight in the model fitting. This might make a good fit with an exponential function even less likely, because even a few events of exceptionally long duration (expected in a power law, and not in an exponential distribution) would influence the fitted line. Therefore, constant bin widths would be the more conservative test for a good exponential fit to power-law distributed data.

Fits that violated our biologically imposed constraints (positive A values and positive τ values) were discarded. We implemented the fitting routine in two different ways. We first considered 1000 consecutive sample datasets, and the probability of rejecting the exponential fits refers only to the subset of 1000 for which exponential fitting converged within our constraints. Fitting with the sum of three exponentials is more likely to include a component that violates our constraints. If there were a systematic relationship between non-convergence and the distribution of bout lengths in the sample, then the results for the three-exponential function would have more of these samples excluded, confounding comparison between the results for one, two and three exponentials. To address this potential bias, we also analyzed the first 1000 samples for which all three exponential functions converged according to our criteria.

The fitted probability mass functions were normalized in order to represent them as fitted probability density functions (PDFs). These PDFs were then used to generate random values distributed according to the fitted exponential functions with the R implementation of the Unuran universal number generator (http://cran.r-project.org/web/packages/Runuran/; accessed 1/12/10) in order to generate the simulated data sets for the KS test (that is, generation of a distribution of *d_s_* values). We investigated a range of sample sizes: *n* = 40, *n*  = 160, and *n* = 640. This range was chosen to parallel approximately the number of sleep-wake transitions observed in a single night (40 or fewer), and to compare with the number of transitions that might occur with pathological fragmentation and/or multiple nights of observation.

## Results

Although several groups have reported mono-exponential fitting to observed bouts of sleep across species, we have recently demonstrated that the distribution of human sleep sub-stages (REM and NREM) is not captured by a mono-exponential model. Specifically, two (REM) or three (NREM) exponential terms were required to fit these distributions, suggesting multiple distinct stage transition time scales [2]. This is of potential interest for statistical as well as biological reasons: 1) mono-exponential fitting of multi-exponential distributions is biased towards brief bout lengths, which dominate frequency distribution histograms, 2) long duration bouts may represent more stable (less fragmented) sleep bouts, and 3) the multi-exponential pattern suggests multiple control points, possibly consistent with neuro-anatomic data suggesting that there are multiple wake- and sleep-promoting nuclei in the mammalian brain.

**Figure 1:**
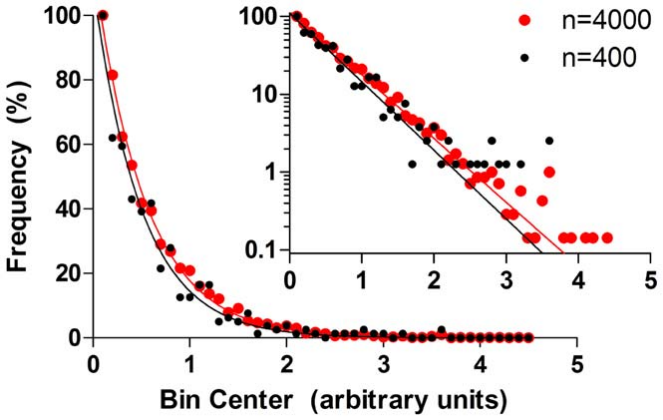
Example of a random fragmentation process. Simple first order Markov transitions between two states (S and W), which generates a one-exponential distribution of sleep states (when n = 400 or 4000). The data are plotted as a binned frequency histogram with arbitrary units of bout duration; inset shows the same data on a semi-log plot, in which mono-exponential distributions appear linear.

The rules governing the timing of sleep-wake stage transitions remain unknown. Given the potential clinical importance of fragmentation (mainly attributed to brief transitions to wakefulness that interrupt sleep continuity), characterizing these patterns empirically from hypnogram data is worthwhile. Consider a simplified model of sleep architecture consisting of two states (sleep and wake) with fixed transition probabilities (a first order markov process). In this setting, the distribution of sleep (and wake) bout lengths is predicted to be mono-exponential. Figure 1A illustrates simulated transitions between a single sleep state and a single interrupting wake state, which results in mono-exponential dynamics as expected. Several groups have reported sleep bout duration to follow an exponential distribution [3], [4], [6].

**Figure 2:**
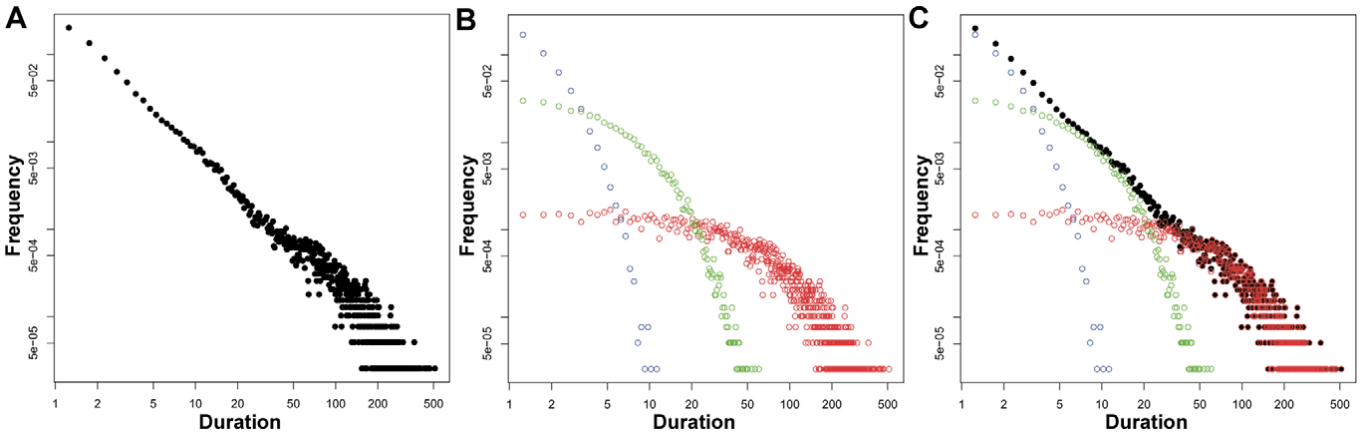
Multi-exponential data can mimic a power law. This frequency histogram appears linear on the log-log plot, which is typical of a power law distribution. The distributions of the three distinct one-exponential generators used to create the distribution shown in panel A include a fast (blue), intermediate (green) and slow (red) exponential decay constant, in arbitrary units of duration. Panel C shows the overlap of panels A and B.

In contrast to the simple shape of a mono-exponential function, a collection of sleep bouts drawn from a multi-exponential distribution can appear linear on a log-log plot, a feature typically considered characteristic of a power law distribution. This is shown in the log-log frequency histogram of Figure 2A. This distribution was formed by three distinct mono-exponential distributions, which are shown separately in Figure 2B. For visual comparison, panels A and B are overlaid in Figure 2C, demonstrating how the three components combine to mimic a power law distribution. Here we specifically chose the parameters of the three exponential components such that the overall distribution would appear linear on a log-log frequency-duration histogram (τ values are 1, 6, 60; relative number of observations are 47.4%, 34.2%, 18.4%; total observations = 38000). To determine the parameters supporting such a similarity between power law and multi-exponential distributions, we simulated sleep bouts using systematic combinations of sample size and parameter values spanning one-, two-, and three-exponential processes.

### KS approach: power law fitting of samples drawn from a combination of two exponential distributions

We conducted simulations to answer the following question: over what range of parameters might a two-exponential process be reasonably fit by a power law function? Thus, we generated sample data sets by varying the exponential decay constant (τ) and the relative proportion of two independent exponential generators. We also varied the total number of observations to determine the impact of sample size on model fitting; these values approximate a single night (40) or multiple nights (160, 320, 640) of stage transitions typical of standard human polysomnograms. In each simulation, the fast τ was fixed at 1 epoch, and the value of the second (slow) τ varied from 2 to 60 epochs (*y*-axis). Frequency-duration histograms of the simulated bouts were subjected to fitting by a power law model, which in this case of exponential data, is by definition “incorrect”. The relative proportion of the faster τ_1_ is given on the *x*-axis (and refers to the number of draws from the fast τ_1_ generator). Therefore the degenerate cases of a single exponential process are shown on either extreme of the *x*-axis (when the relative proportion of the fast τ_1_ events is either 0 or 1). The *z*-axis is the color-scaled probability that the KS routine failed to reject the wrong model, in this case, a power law model fit to the double exponential generated data. A value of zero (green) means the power law fitting was always rejected, while a value of 1 (red) means that the power law was never rejected. Thus, the *z*-axis is a measure of the extent to which the exponential distributions can mimic a power law.

**Figure 3:**
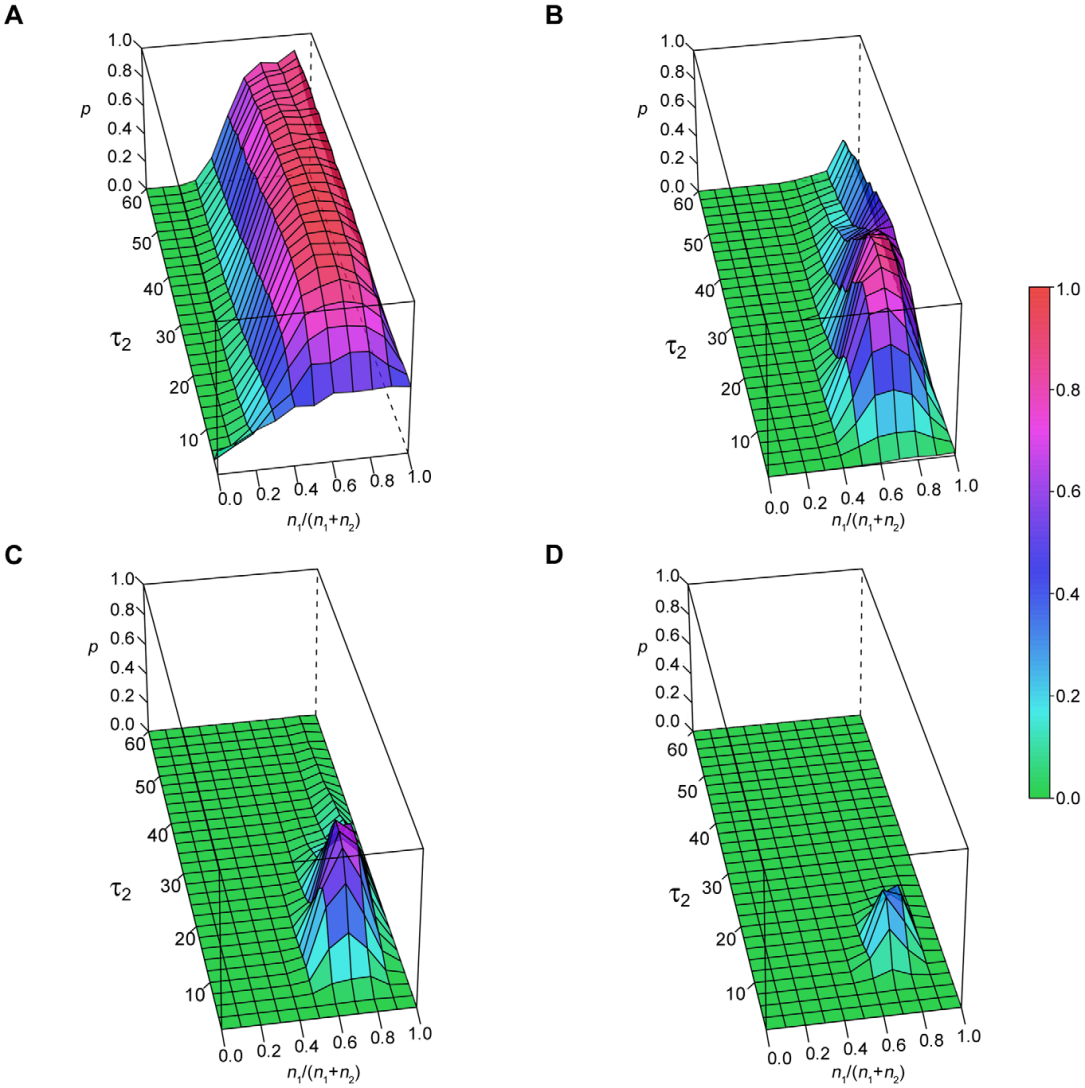
Fitting a power law model to two-exponential data. Panels A–D show the probability of failing to reject the incorrect power law model, for two-exponential data of increasing sample sizes (*n* = 40–640), In each panel, τ_1_ = 1 (the fast exponential), τ_2_ is varied (*y*-axis; the slower exponential), and the relative contribution of each exponential τ is given on the *x*-axis. The probability of rejecting the power law fit is color coded on the *z*-axis.

**Figure 4:**
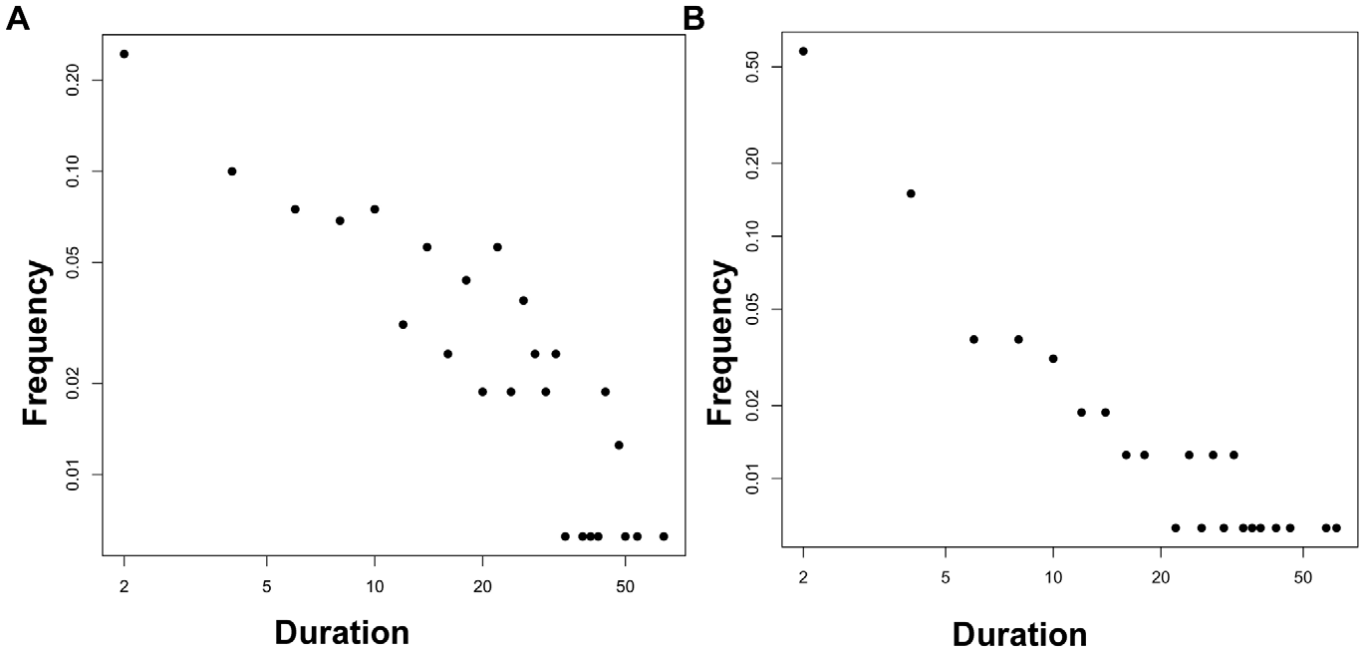
Representative data sets from two-exponential data. Frequency histogram examples of a single trial from the “green” zone of Figure 3B (left) and from the red zone (right). In the green zone, the power law is rejected, while in the red zone, the power law model is acceptable (fails to be rejected).

For *n* = 40 observations (Figure 3A), as the contribution of the fast τ component increases, the probability that a power law model is not rejected increases, reaching a plateau near relative proportions of 0.6 to 0.9, followed by a sharp decline as the proportion approaches 1. This general pattern holds for a range of values of the slow τ_2_ when *n* = 40 (that is, the zone of mimicry is broad). However, when the number of observations is increased to 160 (Figure 3B), the main region in which KS fails to reject the power law model is limited to when τ_2_ is between ∼10–30 epochs. When the number of samples is increased to 320 (Figure 3C), a further decrease in mimicry is seen, and mimicry is negligible when n = 640 (Figure 3D). These results emphasize the importance of sample size when estimating model goodness-of-fit at these relatively small but clinically relevant sample sizes. Example distributions from the green (power law rejected) and red (power law not rejected) regions of the landscape are shown in Figure 4.

### Ordinary least squares (OLS) approach: power law fitting of samples drawn from a combination of two exponential distributions

**Figure 5:**
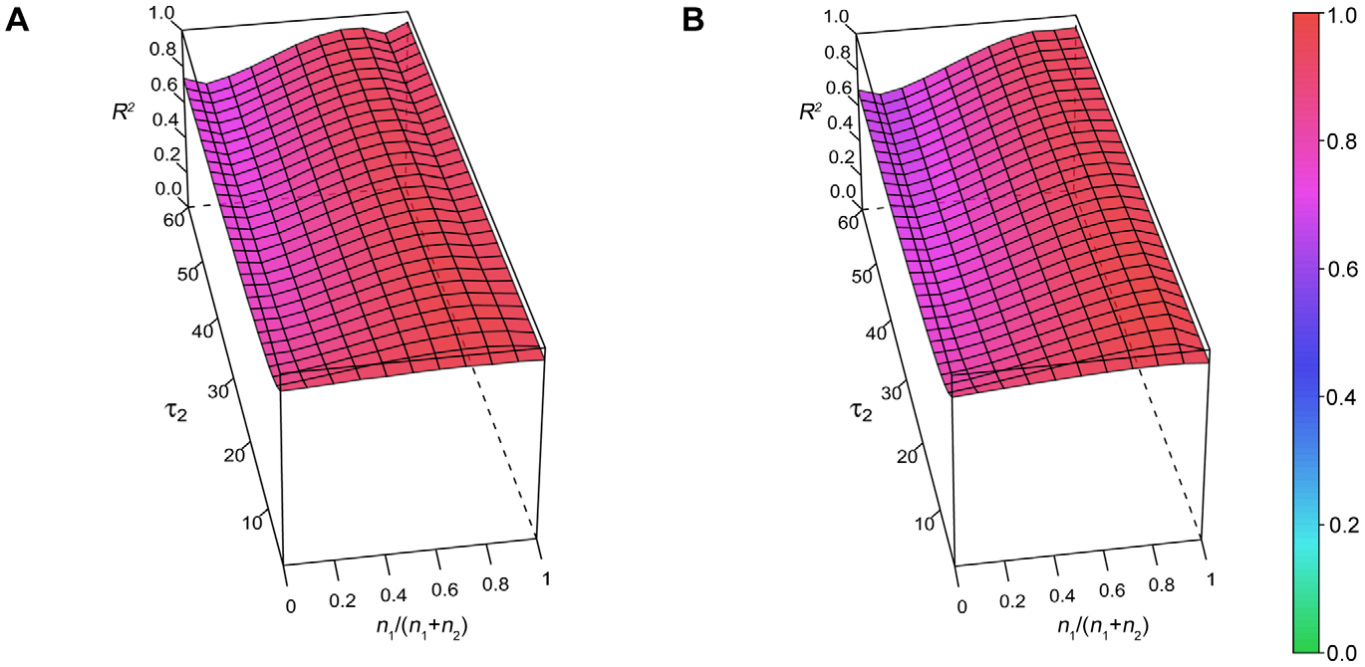
Ordinary least squares fitting of power law model to two-exponential data. For *n* = 40 (A) and *n* = 640 (B), the explained variation (*R* ^2^) from the (incorrect) power law model is high for all parameter values when the OLS method is used.

We repeated power law fitting of the same simulations as in Figure 3 using the OLS method to test the goodness of fit (as is typically performed in the literature [3], [8]). Note that whereas the KS method yields a probability that the sample was drawn from a power law distribution, the *R* ^2^ from an OLS analysis measures the amount of variation in the sample that is explained by a power law model. The *R* ^2^ cannot therefore be used to accept or reject a hypothesis via threshold or cut-off values in the same manner that is commonly implemented with a *p-*value. In Figure 5, the results of OLS fitting are shown for the two-exponential parameter space, with the *R* ^2^ value on the *z*-axis, when *n* = 40 (Figure 5A) and *n* = 640 (Figure 5B). The high *R* ^2^ values (red) indicate that the power law model explains most of the sample variation across the entire parameter landscape (the data mimic a power law across the parameter space), despite the distribution being drawn from a two-exponential distribution. Comparing these results to those from the KS test, it is clear that the *R* ^2^ from the OLS approach is not an appropriate measure of goodness-of-fit under these conditions.

### KS approach: power law fitting of samples drawn from a combination of three exponential distributions

**Figure 6:**
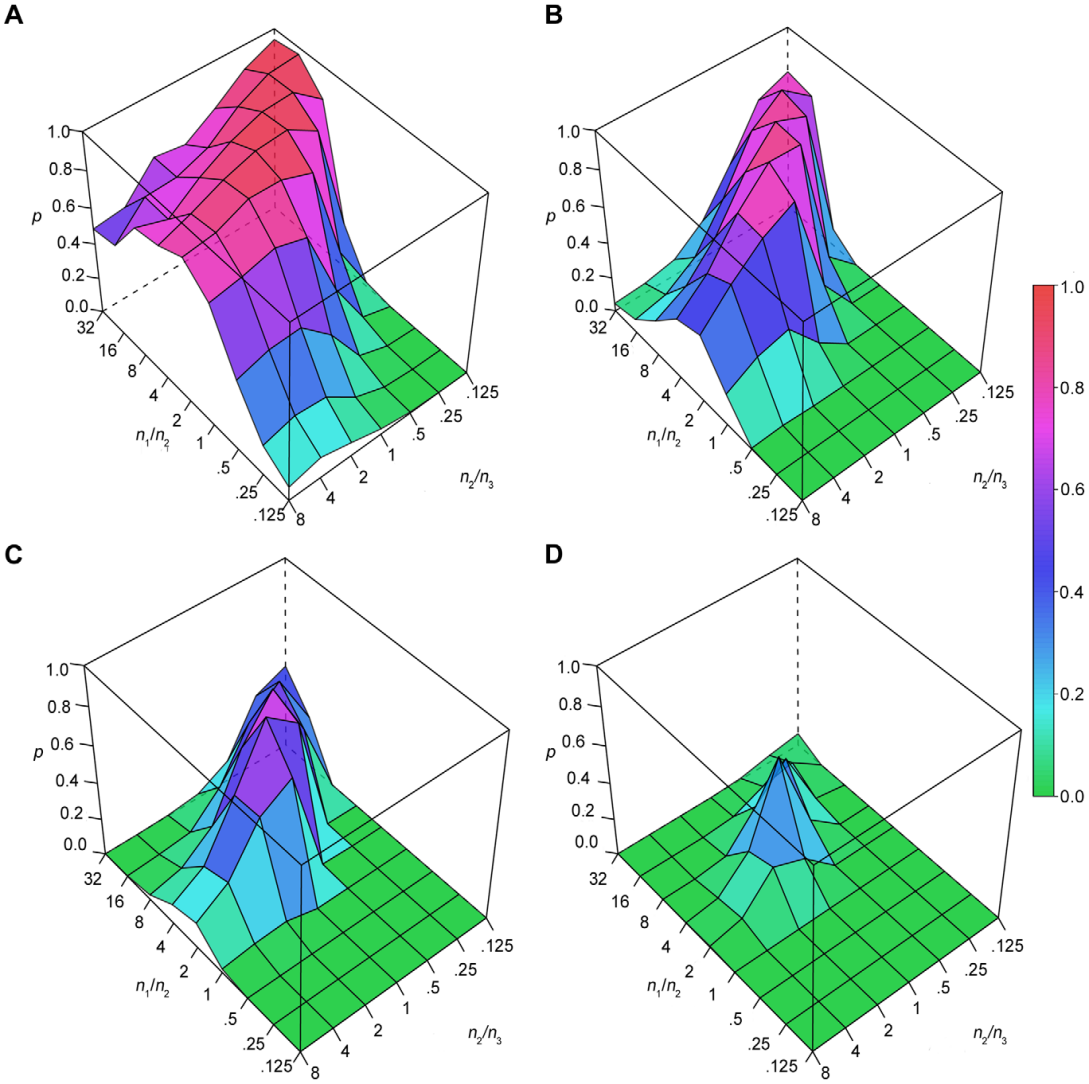
Power law fitting of three-exponential data (fixed τ, variable contributions). A–D show the probability of failing to reject the (incorrect) power law fit of three-exponential data with increasing sample size (*n* = 40–640). The τ values were fixed (1, 5, and 25 epochs), while their proportions are varied as shown by the *x*- and *y*-axes. The probability of failing to reject the power law model is color coded in the *z*-axis.

We next evaluated the range of parameters within which a three-exponential process is well-fit by a power law model. In one set of simulations, we held the τ values for the three exponential functions constant (τ_1_ = 1, τ_2_ = 5, τ_3_ = 25), and systematically varied the proportion of observations drawn from each function. In each case, we evaluated the goodness of fit of a power law model with the KS method. The probability of failing to reject the power law model is shown for *n* = 40 (Figure 6A), *n* = 160 (Figure 6B), *n* = 320 (Figure 6C), and *n* = 640 (Figure 6D) observations. To represent the proportional contribution of all three functions on only two axes, we define the *x* and *y* axes as representing ratios: the *x*-axis shows the ratio of the number of draws from the τ_2_ exponential function to the number of draws from the τ_3_ (slowest) exponential function; the *y*-axis shows the ratio of the number of draws from the τ_1_ (fastest) exponential function to the number of draws from the τ_2_ exponential function. In this manner, all combinations in the parameter space can be visualized. When *n* = 40, the (incorrect) power law model is not rejected throughout most of the parameter space; the power law fit was mainly rejected when the contribution of τ_1_ was low, especially when τ_2_ was also low. As the number of samples increased, the initially broad range of failure to reject became narrower, with a peak occurring when τ_1_:τ_2_ was ∼2–16, and τ_2_:τ_3_ was ∼1–0.25. Like the two-exponential simulations, when *n* = 640, there was minimal chance of failing to reject the power law model.

**Figure 7:**
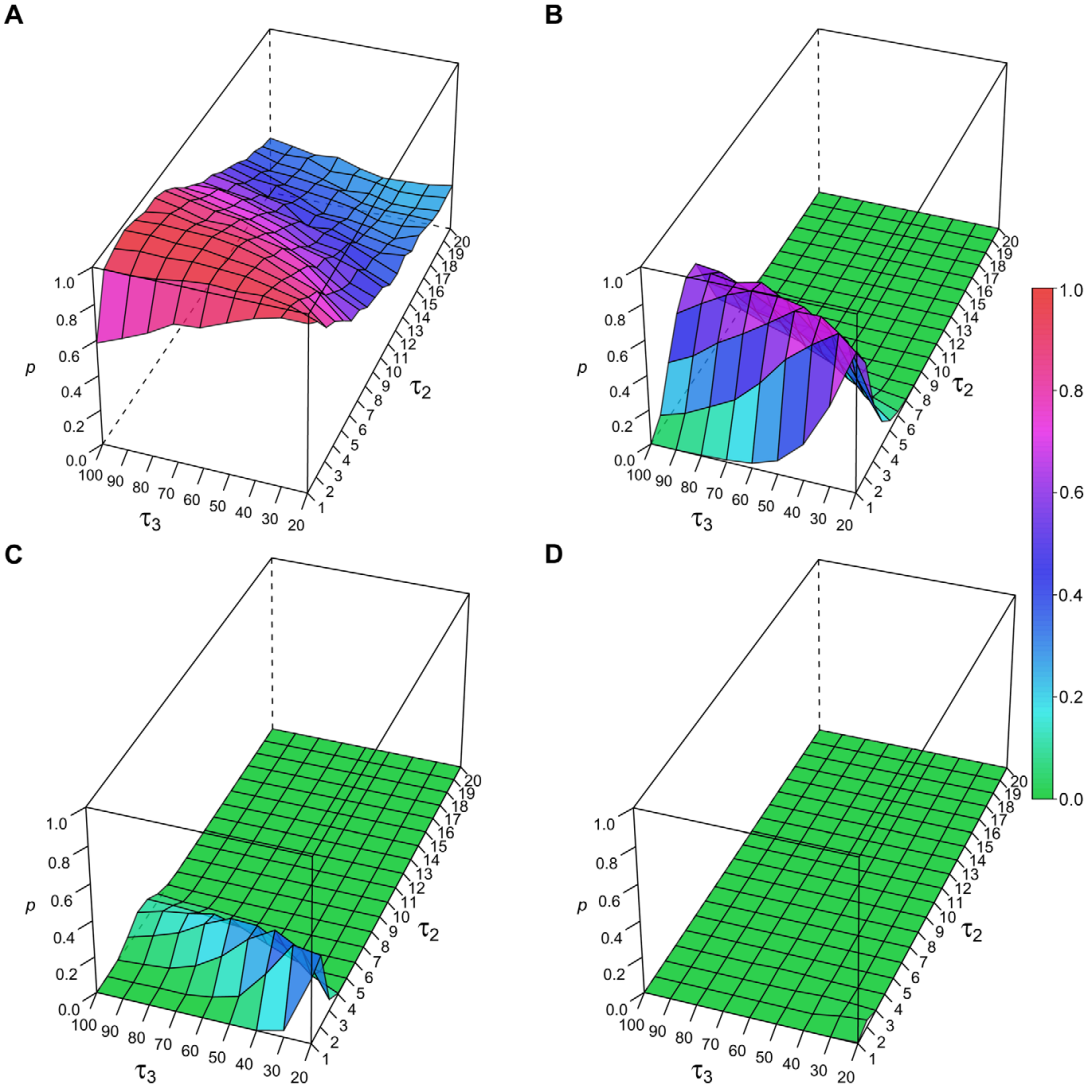
Power law fitting of three-exponential data (fixed contributions, variable τ). A-D show the probability of failing to reject the (incorrect) power law fit of three-exponential data with increasing sample size (*n*=39-639). The τ values are varied as shown by the *x* and *y*-axes. The proportion is fixed at 1∶1∶1. The probability of failing to reject the power law model is color coded in the *z*-axis.

In a complementary set of simulations, we held the number of draws from each exponential function constant and in equal proportions (1∶1∶1). We varied instead the decay constants for the middle (τ_2_) and slowest (τ_3_) decaying exponential functions, while keeping τ_1_ fixed at 1. The probability of rejecting the power law model is shown when the total number of observations was *n* = 39 (Figure 7A), *n* = 159 (Figure 7B), *n* = 318 (Figure 7C), and *n* = 639 (Figure 7D). As expected, failure to reject the power law model was most apparent when *n* = 39; in this condition, the highest probability of failing to reject the power law fit was seen when τ_2_ was between ∼2–10 epochs, and was fairly insensitive to changes in the value of τ_3_. When n = 159, the zone of failure to reject the power law was concentrated around τ_2_ values of ∼2–5, again fairly insensitive to the values of τ_3_. For larger sample sizes, there was minimal chance of failing to reject the power law model.

### Fitting exponential functions to samples drawn from a power law distribution

We next performed simulations to answer the converse question: given a known power law distribution of data, what is the likelihood of incorrectly accepting exponential fitting? To accomplish this, we generated random draws from a power law distribution, and determined the probability of rejecting one-, two-, or three-exponential model fits to the data, using the KS method (see methods). We evaluated total sample sizes of *n* = 40, *n* = 160, and *n* = 640. We performed this analysis with two different criteria related to convergence of the fitting algorithm. In the first, we analyzed 1000 simulated data sets, and included the result in the calculation of probability of rejecting the exponential model only when the fitting routine converged for each exponential model (that is, a subset of the 1000 simulated data sets). In the second, we continued running simulations until all three exponential models converged for a total of 1000 data sets, to avoid potential bias introduced by failure to converge (that is, more than 1000 simulations were required). As reported in Table 1, the probability of rejecting the incorrect exponential models was similar using each of these two criteria.

**Table 1:** Fraction of samples for which exponential fitting is not rejected.

	*n = *40		*n = *160		*n = *640	
one exponential	0.900	(448)	0.127	(914)	0.000	(1000)
	0.882	*	0.141	*	0.000	*
two exponentials	1.000	(405)	0.980	(904)	0.549	(999)
	1.000	*	0.972	*	0.542	*
three exponentials	1.000	(377)	1.000	(884)	0.828	(996)
	1.000	*	0.999	*	0.826	*

Note: a number in parentheses after the result indicates the number of data sets out of the first 1000 simulations that fit our inclusion criteria.

*indicates that the reported result is based on 1000 total data sets for which the one-, two- and three-exponential models all fit our inclusion criteria.

Surprisingly, even the mono-exponential model cannot be rejected in a substantial portion of trials if the power law distribution is under-sampled (*n* = 40), while increasing to *n* = 160 leads to 90% rejection levels, and *n* = 640 leads to nearly 100% rejection. The two- and three-exponential models were acceptable in nearly all of the under-sampled (*n* = 40 and *n* = 160) trials. Even when *n* = 640, the three-exponential fitting was not rejected in over 80% of trials. This suggests some asymmetry of the goodness of fit testing: whereas increased sample size minimizes incorrect power law fitting of a multi-exponential process at sample sizes of *n* = 640 (Figures 3,6,7), power law data could still be incorrectly fitted with multi-exponential functions in a majority of trials due to the larger number of free parameters (Table 1).

## Discussion

Measurements that characterize sleep architecture according to state transition dynamics may capture the elusive concept of sleep continuity (or fragmentation) better than routine clinical statistics such as stage percentages or sleep efficiency. Analysis of sleep-wake state transition probabilities in animal and human studies suggests that the temporal stability of certain stages is approximated by either an exponential or a power law model. Our results emphasize that the distinction between a power law and multi-exponential process is not always straightforward – visually or statistically. By simulating sleep bout lengths based on a variety of known distributions (exponential or power law), we determined goodness of fit by the KS method for the incorrect model: power law for a known exponential distribution, and exponential for a known power law distribution. The parameter landscape under which the incorrect model provided a good fit of the data (that is, “zones of mimicry”) corresponded closely to the τ values for multi-exponential fitting of wake and NREM sleep bouts observed in our analysis of Sleep Heart Health Study subjects [2]. For example, the τ values for wake bouts were approximately 0.6, 3, and 14 epochs, and those of NREM bouts were approximately 2, 8, and 44 epochs.

Several practical challenges exist regarding the quantification of sleep architecture dynamics. Our study does not directly address whether the power law or multi-exponential model is appropriate for any given experimental dataset (where the true distribution is not known). Importantly, our results show that the commonly used OLS fitting method [3], [8] is not suited to measuring goodness of fit of power and multi-exponential models when the underlying distribution is known, consistent with Clauset et al [14]. Two other practical challenges involve sample size and accuracy of identifying state transitions experimentally. Given the difficulty in obtaining multiple nights of PSG data from individual patients, it is particularly important to recognize that the zones of mimicry are highly sensitive to sample size, and thus fitting of clinical PSG data should be approached with caution.

Brief transitions are subject to inter-rater variability in manual scoring and to “rounding” criteria in scoring guidelines. Since brief transitions contribute not only to the steep decay portion of the frequency-duration histograms, but also to the tail portions (by interrupting otherwise long bouts), these fitting methods may be particularly sensitive to accurate determination of brief transitions. Pooling clinically similar subjects may address the sample size challenge, but introduces uncertainty in terms of inter-individual heterogeneity and therefore in the observed statistical distribution of the data. Longitudinal home monitoring of sleep-wake stages is not currently available.

It has been suggested that sleep-wake architecture resembled the dynamics seen in some models of self-organized criticality: in avalanche models, the duration of the avalanche events followed a power law (and was thus likened to wake durations), while the time between avalanches followed an exponential distribution (and was thus likened to sleep durations) [4]. This interpretation has the appeal of reflecting distinct dynamics regulating sleep and wake transitions, and may also reflect species-specific sleep stability that may relate to metabolic factors (although the analysis of sleep across species remains controversial [16], [17]). However, in studies reporting power law dynamics of wake distributions, fits were limited to the linear-appearing portions of complex frequency-duration histograms [4], [8]. An alternative interpretation is that a multiple exponential model accounts for the entire distribution of the frequency duration histogram. Therefore, a systematic approach toward empiric data for which the true generator process is unknown, such as sleep and wake bout distributions, should be considered. Because of the possible relevance of the longest (stable) and shortest (fragmentary) duration sleep or wake bouts to the restorative properties of sleep, we suggest that the entire distribution of state durations should be represented by modeling methods. Although some have postulated time-varying or semi-Markov models for sleep-wake architecture, a simple first order time invariant Markov model might also account for the complexity of empiric sleep-wake distributions [18], [19], [20]. It is also interesting to consider that a system of exponential generators could interact in a manner that would produce power law-like dynamics [21]. For example, Blumberg et al argued that the immature rodent brain exhibits exponential dynamics that evolves into power law dynamics in the adult [9]. Interestingly, Bernstein's theorem [22], an application of the Laplace transform, states that any purely monotonic function can be expressed as a sum of exponentials. Since power law models exhibit a monotonic distribution, it is perhaps not surprising that such a process could be well-described by a sum of exponential decay functions. The converse is that a sum of exponential functions can appear to be power-law-like.

The implication for sleep, and indeed perhaps any setting in which random processes may self-organize, is that asking whether a distribution is *either* exponential *or* a power law may be less informative than asking about the specific organization of component exponentials, how it occurs, and how it can be disrupted in disease. For example, our simulations clearly demonstrate certain combinations of decay time and proportion are best at producing power-law-like patterns. This may have implications for the orchestration of physiological processes, each of which may be fundamentally exponential, but may coordinate into power law dynamics. Future research could test this hypothesis that component exponential processes organize into a power law in physiological systems such as sleep-wake timing (for example, the developmental evolution reported in [3]). An extension of such a hypothesis is that certain sleep pathologies may be related to disruption of such coordinated behavior.

Our current study focused on the question of model fitting, and raised cautionary insights about certain distributions mimicking one another from a fitting standpoint. The related question of model choice is also of interest, but not directly addressed by our analyses: given an empiric set of observations, which model is more likely to explain the data. This question is best undertaken with the guidance of (preferably strong) *a priori* reasons to postulate the expected or “true” distribution, such as exponential or power-law (and not *both* in the sense of multiple exponentials organized into a power-law). Unfortunately, there are reasonable arguments to consider power law and multiple exponential models for the distribution of sleep-wake duration distributions. Moreover, the models are not nested and in fact have quite distinct parameters, and therefore model choice is not straightforward. Clauset et al. recommended Vuong's likelihood ratio test for evaluating non-nested hypotheses of model choice [14]. This test considers the likelihood ratio between competing models for each data point in the distribution. However, as might be surmised from our current study of model fit, the process of model choice is not always accurate. We performed Vuong's test to choose between power law and a 3-exponential model for a known 3-exponential distribution taken from a zone of mimicry (τ values of 1, 5, and 25, with a ratio of draws of 4∶1∶0.5; total *n* = 640). Vuong's test incorrectly concluded that the power law distribution was a better fit in 1000 out of 1000 synthetic data sets. This error in model choice was due to the preponderance of the fast phase of exponential decay in this zone, combined with the relatively low numbers of total draws (relative to the asymptotic need for large data sets for optimal model choice). To demonstrate the relative importance of the fast phase (over sample size), Vuong's test correctly distinguished a slower decaying exponential distribution as exponential rather than power law.

Many other processes in the biological sciences have been analyzed in terms of a power law distributions and their alterations in disease [11], [23], [24], ranging from electroencephalography [25], to actigraphy [13], heart rate variability [26], and gait [27]. In certain settings, the underlying biology of a system may be understood well enough to suspect one or the other distribution *a priori*, with the understanding that there are multiple settings in which power law behavior may be generated [21]. However, as we describe above, for sleep-wake transitions, the expectation of any particular distribution is less straightforward. We suggest therefore that the question of which model (power law or multi-exponential) is better or correct (which implies that they are mutually exclusive) should be weighed against the possibility that an appropriately scaled multi-exponential process is actually a mechanism by which power law behavior can be produced.

In conclusion, we suggest that the two fundamental aims of sleep architecture analysis are 1) to provide a “top-down” approach to mirror the extensive “bottom-up” approaches to sleep-wake mechanisms and physiology, and 2) to provide improved clinical metrics of normal sleep and its disruption in disease states. Applying these methods to sleep-wake dynamics of animals with anatomical lesions or pharmacological manipulations of the critical pathways [10] may facilitate mechanistic understanding of these distributions, and possibly tease apart the multi-exponential versus power law discussion. Although the question of which model is statistically “correct” remains open to further analysis, we raise the question of whether in fact both models are biologically relevant in that a system of exponentials behaves like a power law. Clearly sleep fragmentation affects physiology and symptoms in complex ways; advancing our ability to quantify and sub-type patterns of fragmentation holds the promise for improved diagnostics and rational interventions.

## Supporting Information

Figure S1 Summary of K-S method for goodness-of-fit. A. Sample data set plotted as a frequency-duration histogram. B. Data from panel A re-plotted as a cumulative probability distribution, along with fitted curve (see methods). The maximum vertical distance between the data and the fitted curve is computed. C. Generated new data set (green) by random number generator defined by the fitted function. The maximum vertical distance between the new data set and the fitted curve is ds. D. Repeat process in panel C 100 times to generate a distribution of ds values.
(0.34 MB TIF)
