# Supplementary figures and images for: Power Law versus Exponential State Transition Dynamics: Application to Sleep-Wake Architecture
Source: PLoS One. 2010 Dec 2;5(12):e14204. doi: 10.1371/journal.pone.0014204 (PMC2996311; doi:10.1371/journal.pone.0014204)

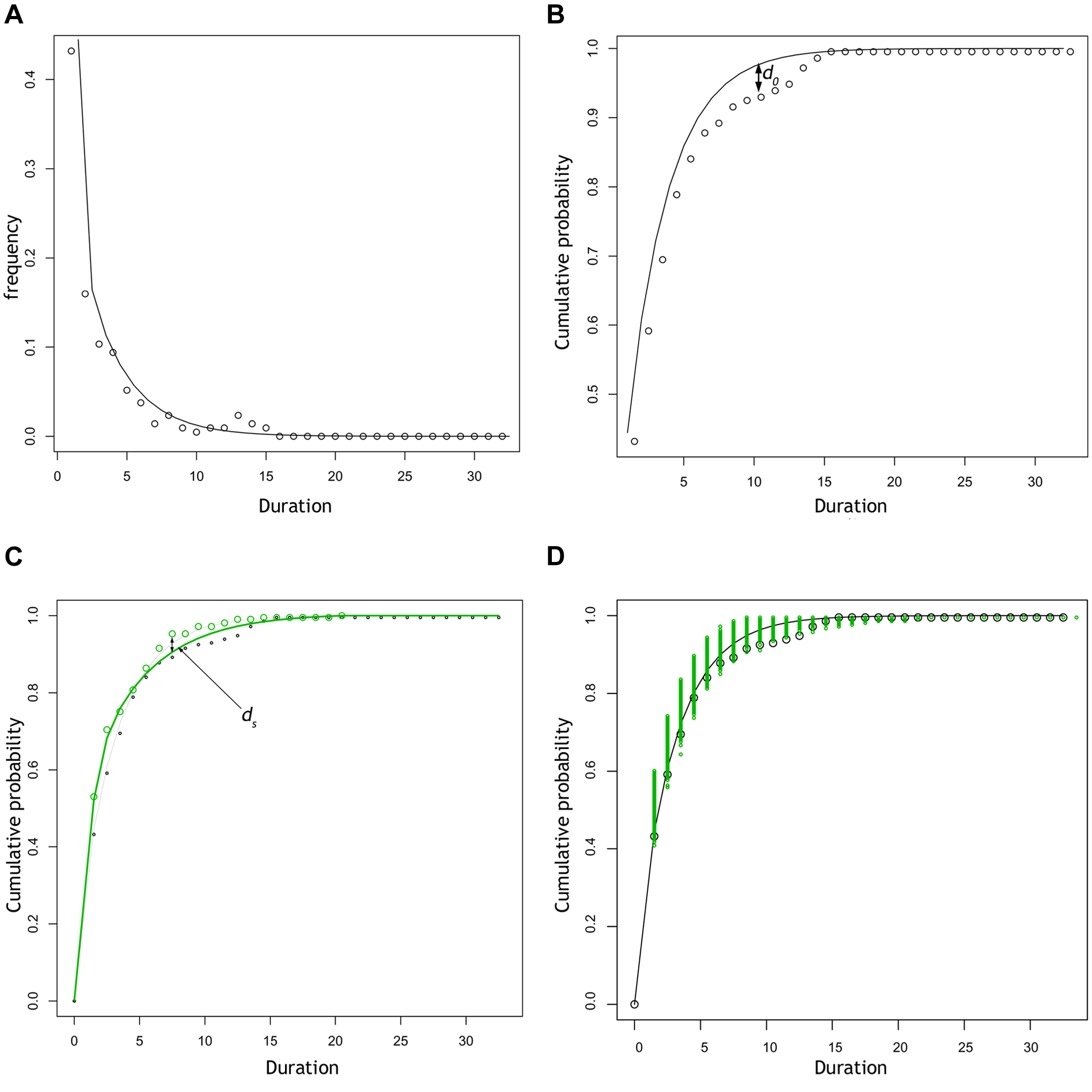

Supplement: Figure S1 — Summary of K-S method for goodness-of-fit. A. Sample data set plotted as a frequency-duration histogram. B. Data from panel A re-plotted as a cumulative probability distribution, along with fitted curve (see methods). The maximum vertical distance between the data and the fitted curve is computed. C. Generated new data set (green) by random number generator defined by the fitted function. The maximum vertical distance between the new data set and the fitted curve is ds. D. Repeat process in panel C 100 times to generate a distribution of ds values. (0.34 MB TIF) [file pone.0014204.s001.tif]
